# A broad diversity in oxygen affinity to haemoglobin

**DOI:** 10.1038/s41598-020-73560-9

**Published:** 2020-10-09

**Authors:** Björn Balcerek, Mathias Steinach, Julia Lichti, Martina A. Maggioni, Philipp N. Becker, Robert Labes, Hanns-Christian Gunga, Pontus B. Persson, Michael Fähling

**Affiliations:** 1 Institut für Vegetative Physiologie, Charité – Universitätsmedizin Berlin, Corporate Member of Freie Universität Berlin, Humboldt-Universität zu Berlin and Berlin Institute of Health, Charitéplatz 1, 10117 Berlin, Germany; 2 Institut für Physiologie, Zentrum für Weltraummedizin Berlin (ZWMB), Charité – Universitätsmedizin Berlin, Corporate Member of Freie Universität Berlin, Humboldt-Universität zu Berlin and Berlin Institute of Health, Berlin, Germany; 3 Department of Biomedical Sciences for Health, Università Degli Studi Di Milano, Milan, Italy

**Keywords:** Physiology, Medical research

## Abstract

Oxygen affinity to haemoglobin is indicated by the p50 value (pO_2_ at 50% O_2_Hb) and critically determines cellular oxygen availability. Although high Hb-O_2_ affinity can cause tissue hypoxia under conditions of well O_2_ saturated blood, individual differences in p50 are commonly not considered in clinical routine. Here, we investigated the diversity in Hb-O_2_ affinity in the context of physiological relevance. Oxyhaemoglobin dissociation curves (ODCs) of 60 volunteers (18–40 years, both sexes, either endurance trained or untrained) were measured at rest and after maximum exercise (VO_2_max) test. At rest, p50 values of all participants ranged over 7 mmHg. For comparison, right shift of ODC after VO_2_max test, representing the maximal physiological range to release oxygen to the tissue, indicated a p50 difference of up to 10 mmHg. P50 at rest differs significantly between women and men, with women showing lower Hb-O_2_ affinity that is determined by higher 2,3-BPG and BPGM levels. Regular endurance exercise did not alter baseline Hb-O_2_ affinity. Thus, p50 diversity is already high at baseline level and needs to be considered under conditions of impaired tissue oxygenation. For fast prediction of Hb-O_2_ affinity by blood gas analysis, only venous but not capillary blood samples can be recommended.

## Introduction

In clinical routine, the oxygenation of patients can be determined in two ways: haemoglobin (Hb) concentration serves to estimate the oxygen (O_2_) carrying capacity of the blood, and pulse oximetry indicates oxygen saturation (sO_2_). However, inadequate O_2_ delivery to the tissue (hypoxia) may occur under conditions of high O_2_ affinity to haemoglobin. In a worst-case scenario, anemia or hypoxemia is combined with a compromised release of oxygen to the cells, enhancing the risk of tissue hypoxia.

Cellular oxygen availability is fundamental for energy metabolism. Alteration of cellular oxygen demand is compensated by adaptation of respiration, cardiac output, and perfusion rate. Tissue oxygen availability is severely disturbed in pathological conditions such as shock, sepsis, pulmonary diseases or coronary heart disease, which are associated with a dysbalance of oxygen consumption and delivery.

Oxygen demand differs between organs and is highest in heart and kidney, followed by brain and liver^1^. Increased activity, e.g. of the skeletal muscle during exercise, requires rapid adaptation of oxygen delivery. Thus, blood flow is regulated according to tissue specific ratio of oxygen delivery (DO_2_) to oxygen consumption (VO_2_)^2^. Under resting conditions, overall oxygen extraction ratio (O_2_ER) amounts to 25–35% only, resulting in a blood oxygen reserve utilized e.g. in chronic anemia. The low oxygen carrying capacity of the blood in anemia is compensated by an increased concentration of 2,3-Bisphosphoglycerate (2,3-BPG, alias 2,3-DPG), an allosteric effector promoting the offloading of oxygen to the tissues by lowering Hb-O_2_ affinity^3^. Thus, O_2_ER can adapt to meet VO_2_^4^ and here, the ability to release the required amount of oxygen to the tissue is of major significance.

Hence, accessing the blood’s oxygen reserve by lowering Hb-O_2_ affinity constitutes a fascinating option to improve tissue oxygenation without any additional metabolic demand for the organism. Increased oxygen off-loading in the tissues has been shown to improve systemic hemodynamics, microvascular function, and exercise capacity^5^. Moreover, lowering Hb-O_2_ affinity offers the possibility of improved tissue oxygenation, while avoiding high blood viscosity and arrhythmias. Consequently, recent therapeutic attempts for hypoxia-related pathologies have targeted Hb-O_2_ affinity exogenous modifiers^6,7^.

Oxygen affinity to haemoglobin was first described by Bohr, Hasselbalch and Krogh (1904), who discovered the S-shaped form of the oxyhaemoglobin dissociation curve (ODC)^8^. Haemoglobin may exist in two alternative structures, the deoxy or T (tense) and the oxy or R (relaxed) structure. Effectors of oxygen binding to haemoglobin modulate the transition between both structures^9^. Temperature and allosteric effectors such as hydrogen ions (pH), carbon dioxide (CO_2_), and organic phosphates, of which 2,3-BPG is the most important, modulate Hb-O_2_ affinity through cooperative effects on haemoglobin^10^. Hb-O_2_ affinity is commonly expressed as the p50 value representing pO_2_ at 50% saturation of haemoglobin with O_2_. Standard p50 in humans is 26.9 mmHg at pH 7.4 and 37°C^11^. Increased values of temperature, hydrogen ions (acidosis), pCO_2_, and 2,3-BPG all lower Hb-O_2_ affinity with higher p50 values and a right shift in ODC. For instance, working muscle elevates local temperature and produces more H^+^ and CO_2_, causing improved oxygen release^12^. The main explanation for the influence of CO_2_ on Hb–O_2_ affinity is the simultaneous change in pH, due to conversion of CO_2_ to bicarbonate via the carbonic anhydrase reaction. Nevertheless, CO_2_ also exerts a specific effect on Hb-O_2_ affinity at constant pH^13^. 2,3-BPG levels are increased in maternal blood providing good oxygen release, while fetal blood shows high oxygen affinity to fetal haemoglobin that is less susceptible to 2,3-BPG^14,15^.

An increase in p50 (lowered Hb-O_2_ affinity) would benefit tissues insufficiently supplied with oxygen. While pH, pCO_2_ and body temperature are tightly regulated, the level of 2,3-BPG might be a useful target. 2,3-BPG is synthesized by Bisphosphoglycerate mutase (BPGM), as described by the Rapoport-Luebering glycolytic shunt^16,17^. Acidosis inhibits and alkalosis promotes BPGM activity, as observed in respiratory alkalosis at high altitude or lactic acidosis during exercise^18–21^. Elevation of BPGM activity at high altitude has been further linked to Sphingosine-1-phosphate levels^22^ and A2B adenosine receptor that mediates phosphorylation of BPGM via the AMP-activated protein kinase^23^. Moreover, a long-term effect of endurance exercise by increasing 2,3-BPG has been reported^24^.

To estimate the impact of Hb-O_2_ affinity on tissue oxygenation and its putative clinical application, it is essential to know the maximum physiological range and individual diversity. This study was designed to address three questions: (i) Is Hb-O_2_ affinity tightly regulated or exists a rather broad diversity in humans at rest that might contribute to individual differences in tissue oxygenation? (ii) Does endurance exercise cause improved cellular oxygen availability by lowering Hb-O_2_ affinity? (iii) Does estimated p50 values from blood gas analysis provide good estimates of Hb-O_2_ affinity? To address these questions, we tested 60 volunteers of both sexes who were either endurance trained or untrained. ODCs were obtained under isocapnic conditions for all participants and VO_2_max test served to define the maximum physiological range of Hb-O_2_ affinity. Strikingly, we observed a marked diversity in Hb-O_2_ affinity that is mainly attributed to sexes and correlates with 2,3-BPG levels and BPGM expression.

## Results

### Definition of cohorts

**Figure 1:**
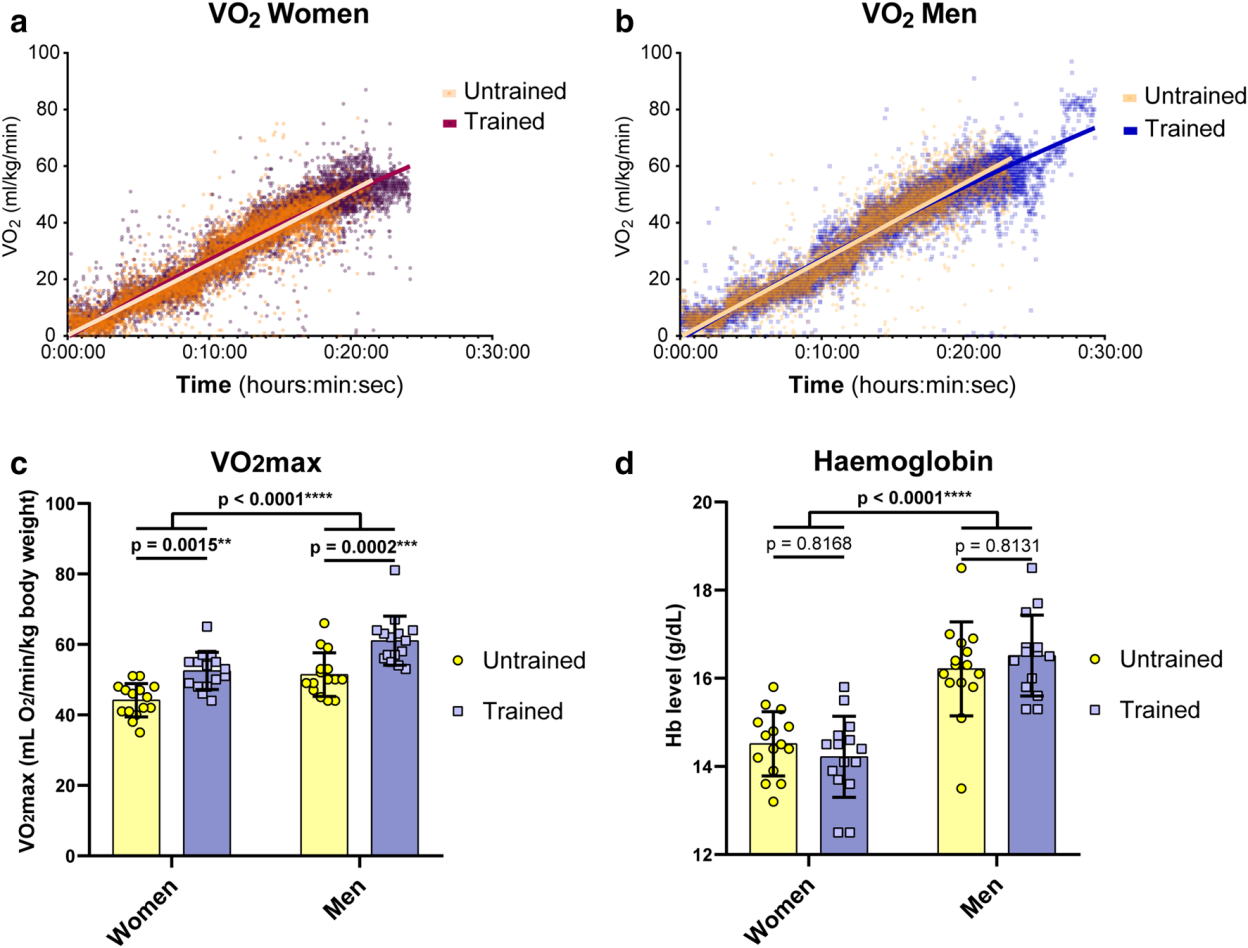
Baseline parameters. 60 volunteers of both sexes and either endurance trained or untrained. (**a**, **b**) VO_2_ values revealed elevated levels in trained groups of women (**a**) and men (**b**). (**c**) Statistics for VO_2_max values comparing either trained versus untrained groups and women versus men. (**d**) Haemoglobin levels by blood gas analysis of capillary probes. N = 15 per group. Two-way ANOVA with post hoc Bonferroni test served to test for significance. Adjusted *p* values are indicated.

As the selection of untrained versus trained groups based on by self-reports, we determined maximal oxygen consumption rate (VO_2_max) as the standard index for fitness level (Fig. 1a–c). We found significantly elevated VO_2_max level in trained versus untrained women (52.47 ± 10.11 vs. 44.13 ± 10.69 mL O_2_/min/kg BW) and men (60.57 ± 11.49 vs. 51.4 ± 12.06 mL O_2_/min/kg BW). Measurements of haemoglobin revealed no differences by fitness level (Fig. 1d), while woman had significantly lower Hb levels compared to men (14.37 ± 0.83 vs. 16.37 ± 0.97 g/dl).

Selected groups showed no significant differences by age, with an average age of 27 years (Supplementary Figure S1a). BMI was slightly lower in women compared to men (22.34 ± 2.22 vs. 23.83 ± 2.42 kg/m^2^), while no differences were found between untrained and trained groups (Supplementary Figure S1b). Muscle mass was higher in trained versus untrained women (44.47 ± 2.57% vs. 39.75 ± 3.35%) with higher body fat in untrained women (19.75 ± 4.58% vs. 27.15 ± 6.01%) (Supplementary Figure S1c, d). Men, however, showed no significant differences between trained and untrained groups in this regard (Supplementary Figure S1c, d). Muscle tissue percentage was lower (42.11 ± 3.79% vs. 48.02 ± 3.76%) and body fat content was higher (23.45 ± 6.46 vs. 15.37 ± 6.32 kg) in female subjects.

Taken together, cohorts indicated group specificity by elevated VO_2_max values because of endurance exercise. Differences in muscle mass, body fat and haemoglobin concentration were as expected regarding fitness level and sexes.

### Estimated (capillary) and calculated (venous) p50 values from blood gas analysis

Blood gas analysis may provide estimated p50_e_ (capillary) and calculated p50_c_ (venous) values from a single blood sample. Capillary blood samples typically reflect the arterial blood gas composition^25,26^ and, thus, estimate p50_e_ from samples of high pO_2_ and oxygen saturation of > 95% in healthy subjects. Venous blood samples show a much greater variation that may depend on haemodynamic and/ or metabolic effects. However, typical venous sO_2_ values lie between 55 and 75%.

**Figure 2:**
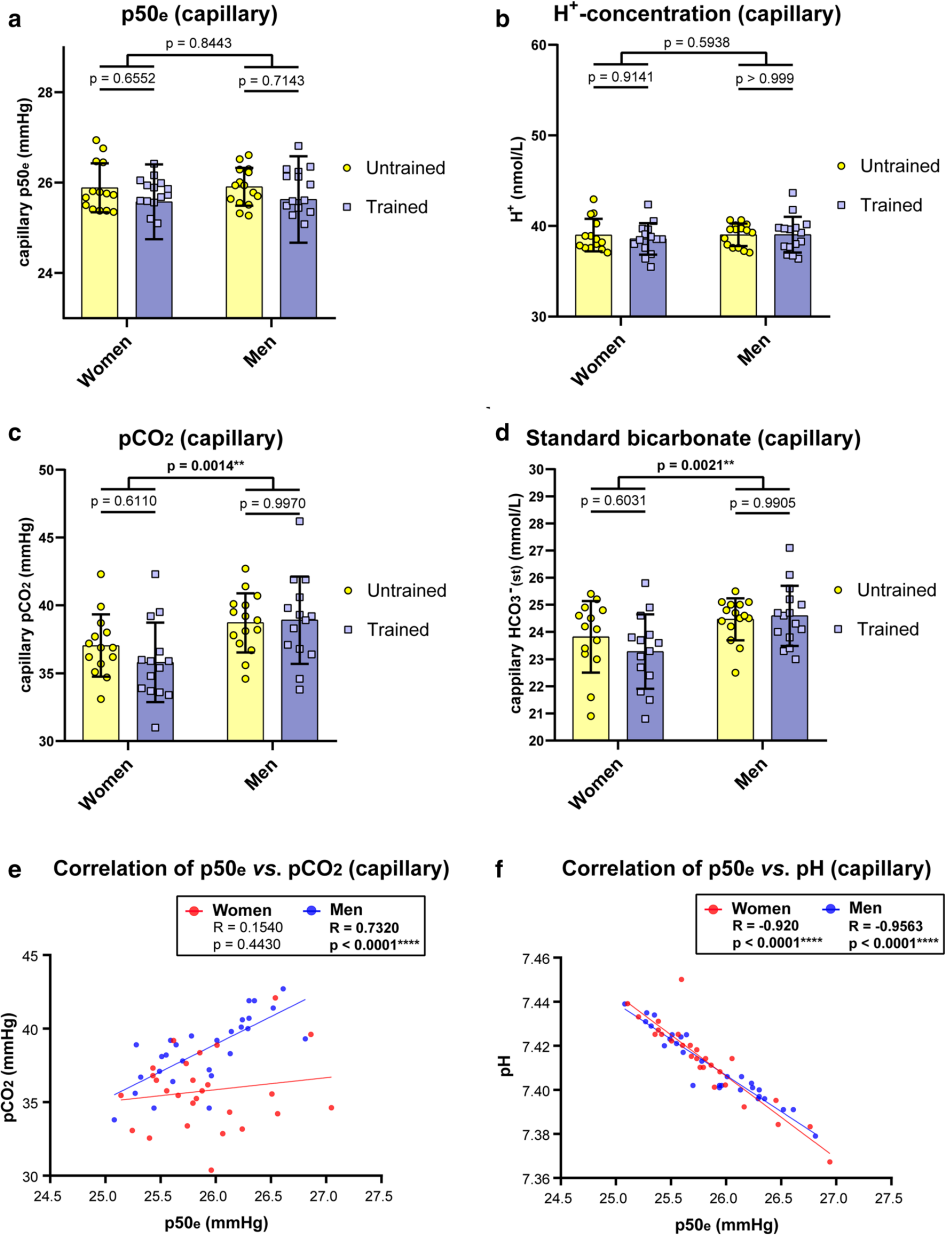
Capillary blood gas analysis. Estimated p50_e_ values (**a**), H^+^-level (**b**), pCO_2_ (**c**) and Standard bicarbonate (**d**) values were obtained from trained versus untrained women and men. Estimated p50_e_ values from capillary probes were correlated with pCO_2_ (**e**) and pH (**f**), both known determinants that affect O_2_-affinity to haemoglobin. N = 15 per group. Two-way ANOVA with post hoc Bonferroni test served to test for significance. Adjusted *p* values are indicated (**a**–**d**). In correlation analysis, the Pearson correlation coefficient R is indicated. Two tailed test with confidence interval 95% served to test for significance (**e**, **f**).

Capillary blood indicated no difference in p50_e_ (mean p50_e_ of all participants: 25.75 ± 0.72 mmHg) (Fig. 2a). Values ranged between 25.08 mmHg (lowest p50_e_) and 26.94 mmHg (highest p50_e_). Capillary pH was identical in all groups (pH: 7.41 ± 0.02) as indicated by H^+^-concentration (Fig. 2b). Notably, pCO_2_ was significantly lower in women (36.42 ± 2.65 mmHg) than men (38.81 ± 2.67 mmHg) (Fig. 2c) that is associated with lower standard bicarbonate (Fig. 2d), causing similar pH levels in women and men. However, under conditions of similar pH and lower pCO_2_, a higher Hb-O_2_ affinity in women would have been expected but was not observed. Interestingly, estimated p50_e_ is correlated with pCO_2_ in men, but not in women (Fig. 2e), raising the question of a sex related miss-estimation. In both sexes, however, estimated p50_e_ indicated an inverse correlation with pH (Fig. 2f).

**Figure 3:**
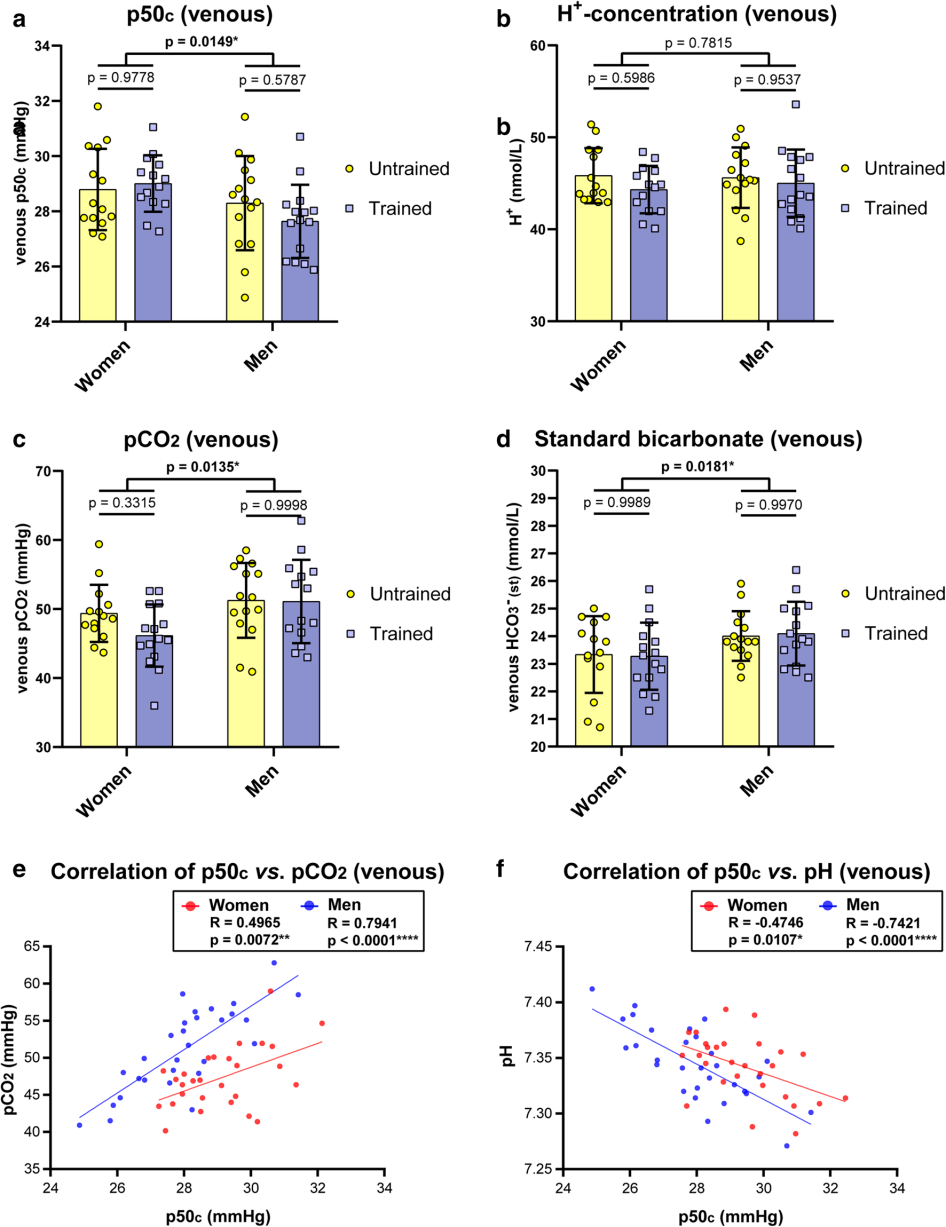
Venous blood gas analysis. Calculated p50_c_ values (**a**), H^+^-level (**b**), pCO_2_ (**c**) and Standard bicarbonate (**d**) values were obtained from trained versus untrained women and men. Calculated p50_c_ values from venous probes were correlated with pCO_2_ (**e**) and pH (**f**). N = 15 per group. Two-way ANOVA with post hoc Bonferroni test served to test for significance. Adjusted *p* values are indicated (**a**–**d**). In correlation analysis, the Pearson correlation coefficient R is indicated. Two tailed test with confidence interval 95% served to test for significance (**e**, **f**).

Venous blood samples showed no difference in calculated p50_c_ values by fitness level, while indicating a significantly higher Hb-O_2_ affinity in men compared to women (p50_c_: women = 28.9 ± 1.25 mmHg vs. men = 27.97 ± 1.54 mmHg) (Fig. 3a). Values ranged between 24.87 mmHg (lowest p50_c_) and 31.8 mmHg (highest p50_c_). Venous pH values were comparable in all groups (7.35 ± 0.03; values were calculated by means of H^+^-levels) (Fig. 3b). Notably, as observed in capillary blood samples, venous pCO_2_ and standard bicarbonate were significantly higher in men versus women (pCO_2_ women: 47.71 ± 4.56 mmHg vs. men: 51.18 ± 5.63 mmHg; HCO_3_^−^ women: 23.3 ± 1.28 mmol/L vs. men: 24.05 ± 1.02 mmol/L) (Fig. 3c,d). Still, higher pCO_2_ should be associated with lower Hb-O_2_ affinity. Therefore, the controversy of higher pCO_2_ level and lower p50_c_ values in men versus women remains open. Nevertheless, venous blood samples showed significant correlations of p50_c_ with pCO_2_ as well as pH for both sexes, while men showed a greater distribution in p50_c_ (Fig. 3e,f).

Taken together, no alteration of oxygen affinity to haemoglobin due to endurance exercise has been observed, neither in estimated (capillary), nor calculated (venous) p50_e/c_ values. Men showed higher bicarbonate levels that are associated with higher pCO_2_ level to maintain a pH similar to women. Notably, between sexes, higher pCO_2_ in men should cause lower Hb-O_2_ affinity, which is not observed. In contrast, calculated p50_c_ values indicated a significantly higher Hb-O_2_ affinity in men compared to women.

### A sex difference in oxygen affinity to haemoglobin

To clarify the issue of p50 prediction, we recorded oxyhaemoglobin dissociation curves (ODC). After equilibration of venous blood, the pH during determination of ODCs was identical in all groups (7.42 ± 0.02), as well as the pCO_2_ (35.25 ± 0.7 mmHg) (Supplementary Figure S2a, b). Of note, the standard pCO_2_ is 40 mmHg for arterial blood and, thus, higher as in our experimental setting, resulting in slightly lower p50 values compared to the literature.

**Figure 4:**
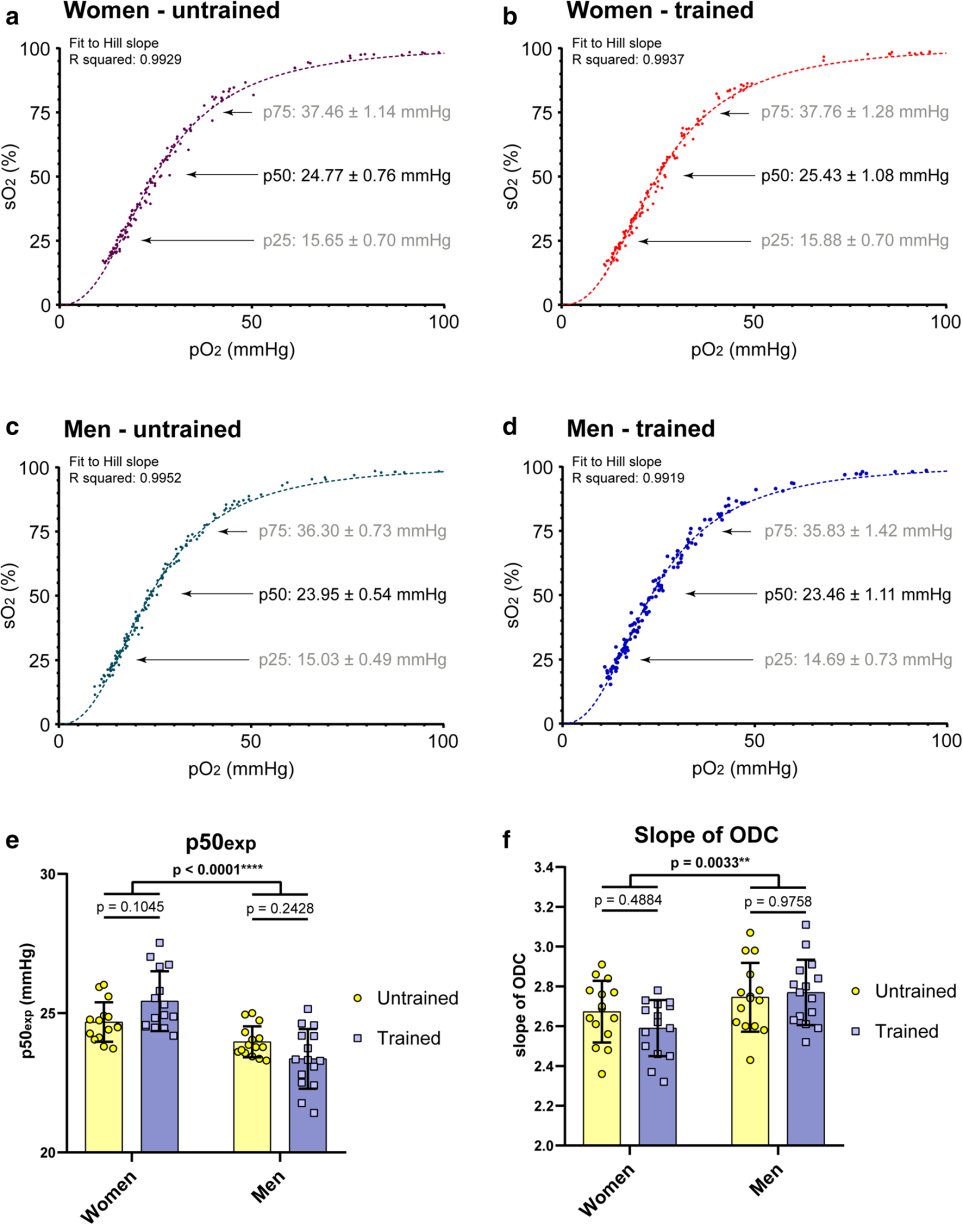
Oxyhaemoglobin dissociation curves (ODC) for trained versus untrained women and men. ODCs of women and men that were either trained or untrained were recorded from venous blood samples and plotted using the “specific binding with Hill slope” model. Data fit to the Hill slope algorithm as shown by R squared values. ODCs shown, depict of 136 (**a**), 149 (**b**), 173 (**c**) and 151 (**d**) data points resulting from 15 single ODCs per group. Oxygen affinity to haemoglobin is indicated by p50 as well as p75 and p25 values. (**e**) Statistics for p50 values indicate no alteration in oxygen affinity to haemoglobin by fitness levels; however, Hb-O_2_ affinity is lower in women than men. (**f**) The Hill slope declines when ODC shifts to the right. The higher affinity of oxygen to haemoglobin (lower p50) in men results in a more precipitous slope. (**e**, **f**) N = 15 per group. Two-way ANOVA with post hoc Bonferroni test served to test for significance. Adjusted *p* values are indicated.

**Figure 5:**
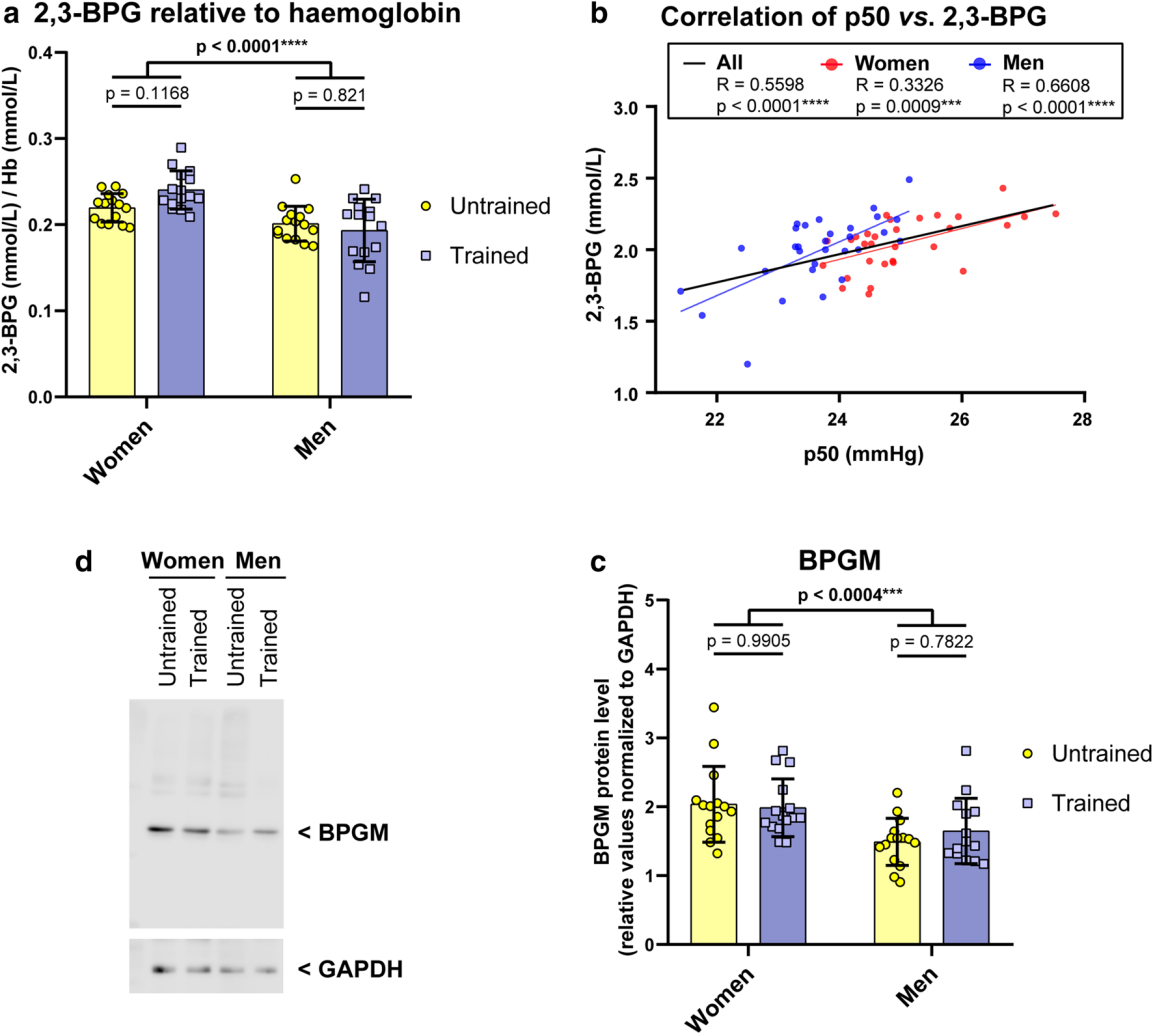
Lower Hb-O_2_ affinity in women compared to men is explained by 2,3-BPG. (**a**) 2,3-BPG levels were normalised to haemoglobin levels, a significant difference was found between sexes, but not fitness levels. (**b**) Correlation of 2,3-BPG level and p50 values revealed significance for women and men as well. The Pearson correlation coefficient R is indicated. Two tailed test with confidence interval 95% served to test for significance. (**c**) Representative Western blot of pooled blood probes to visualise BPGM level. Values were normalised to GAPDH, a glycolytic enzyme that is well expressed in erythrocytes. (**d**) Statistics of BPGM protein level. A and D: N = 15 per group. Two-way ANOVA with post hoc Bonferroni test served to test for significance. Adjusted *p* values are indicated.

To define the Hb-O_2_ affinity regarding fitness level and/ or sexes, we determined p75, p50, and p25 values (Fig. 4). All data sets from single ODCs per group were combined. Resulting ODCs fit to the Hill slope as indicated by R^2^ values of above 0.99 in each group (Fig. 4a–d). In line with venous p50_c_ values, we found no alteration of Hb-O_2_ affinity due to endurance exercise (Fig. 4e). Nevertheless, women showed a significantly lower Hb-O_2_ affinity compared to men, as indicated by higher p50 values (Fig. 4e). Values ranged in a difference of 6.11 mmHg (highest to lowest p50) (Figs. 4e, 5b). After combining the data of untrained and trained participants and calculating the mean ± SD for women and men, we found p75 values of 37.61 ± 1.19 mmHg (women) versus 36.07 ± 1.14 mmHg (men), p50 values of 25.09 ± 0.97 mmHg (women) versus 23.7 ± 0.9 mmHg (men) and p25 values of 15.76 ± 0.7 mmHg (women) versus 14.86 ± 0.63 mmHg (men). Thus, the mean difference of sexes observed is 1.54 mmHg (p75), 1.39 mmHg (p50), and 0.89 mmHg (p25). When comparing the differences of p75, p50 and p25 values, it is obvious that the slope of ODC is lower when shifting to the right, thus, the slope of ODC is steeper in men compared to women (Fig. 4f).

### 3-BPG determines p50 of sexes

As pCO_2_ and temperature were standardised and pH showed no difference between groups, we suggested 2,3-Bisphosphoglycerate (2,3-BPG, alias 2,3-DPG) to be responsible for the sex dependent difference in oxygen affinity to haemoglobin. We found significantly higher 2,3-BPG levels in women compared to men (Fig. 5a), with no differences by fitness level. 2,3-BPG correlated strongly with corresponding p50 values (Fig. 5b), which also holds true when correlating values of women and men separately.

Differences in 2,3-BPG level might either result from differences in BPGM expression level or BPGM activity. As the only known effector of BPGM activity is blood proton (H^+^) level (low pH is blocking BPGM activity)^19,21^, and pH values were similar in our groups, we determined BPGM protein level (Fig. 5c,d). The measurements revealed that lower 2,3-BPG level is, indeed, associated with lower BPGM expression in men compared to women.

### No limitation of cellular oxygen availability in men during exercise

**Figure 6:**
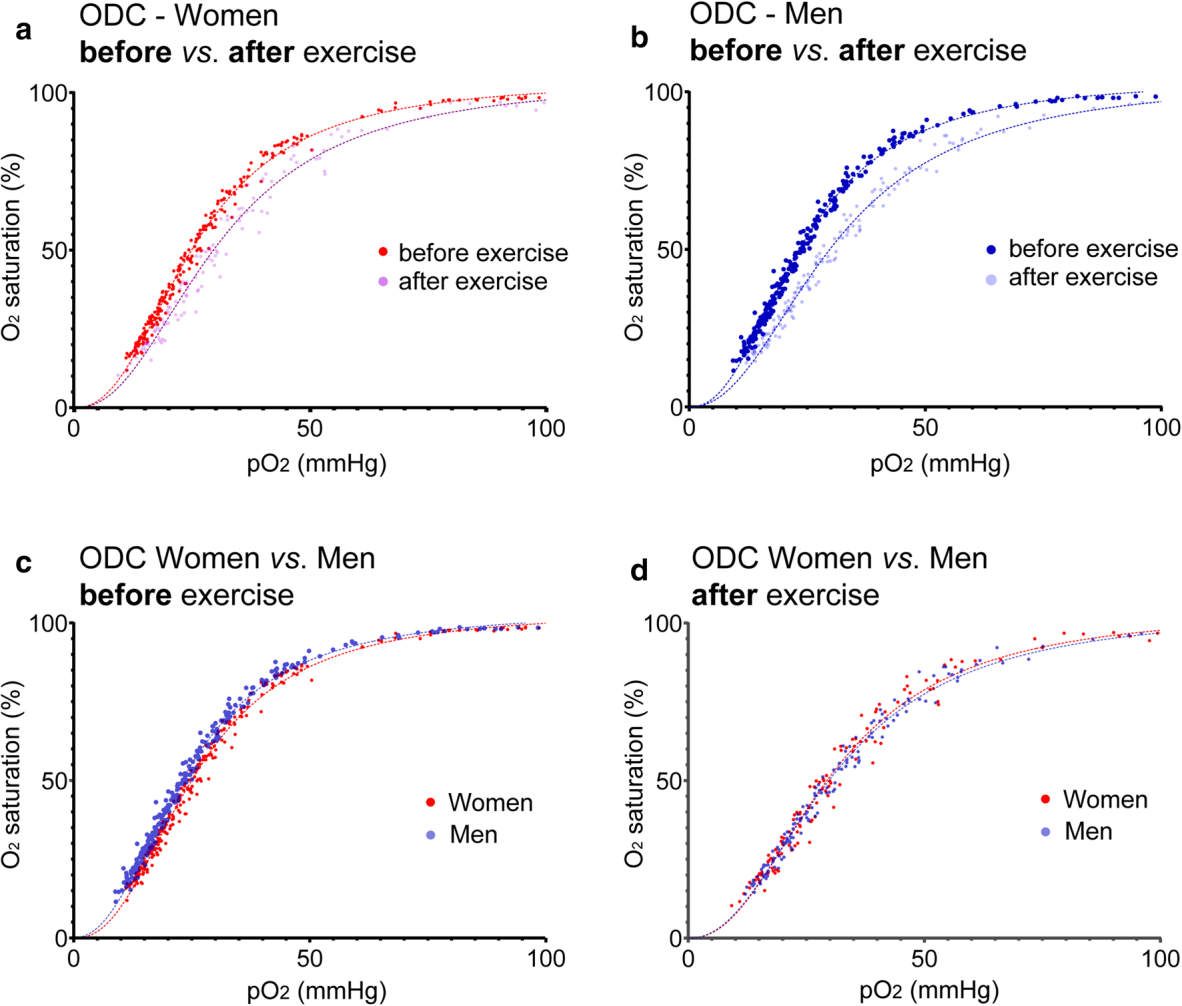
Oxyhaemoglobin dissociation curves (ODC) in women versus men before versus. after VO_2_max test. Individual venous blood samples were used to monitor the ODCs by tonometry under isocapnic conditions at 37 °C. Measurements were performed after arterialisation of blood using carbogen gas (95% O_2_, 5% CO_2_) followed by deoxygenation (95% N_2_, 5% CO_2_) up to a blood oxygen saturation of lower than 20%. N = 30 women and 30 men before exercise; N = 15 women and 15 men after exercise.

The observed range of individual p50 values per se, warranted further investigation to estimate its impact. Thus, we recorded ODCs after VO_2_max test and focussed on the sex difference. ODCs are presented for women and men before versus after exercise (Fig. 6a,b) as well as women versus men before and after exercise (Fig. 6c,d). The maximum right shift of ODC under physiological conditions is indicated for women (Fig. 6a) and men (Fig. 6b). It is important to note that the right shift of ODC after exercise (shown in Fig. 6a,b) is solely attributed to the alteration of pH, as the pCO_2_ and temperature were kept stable.

**Figure 7:**
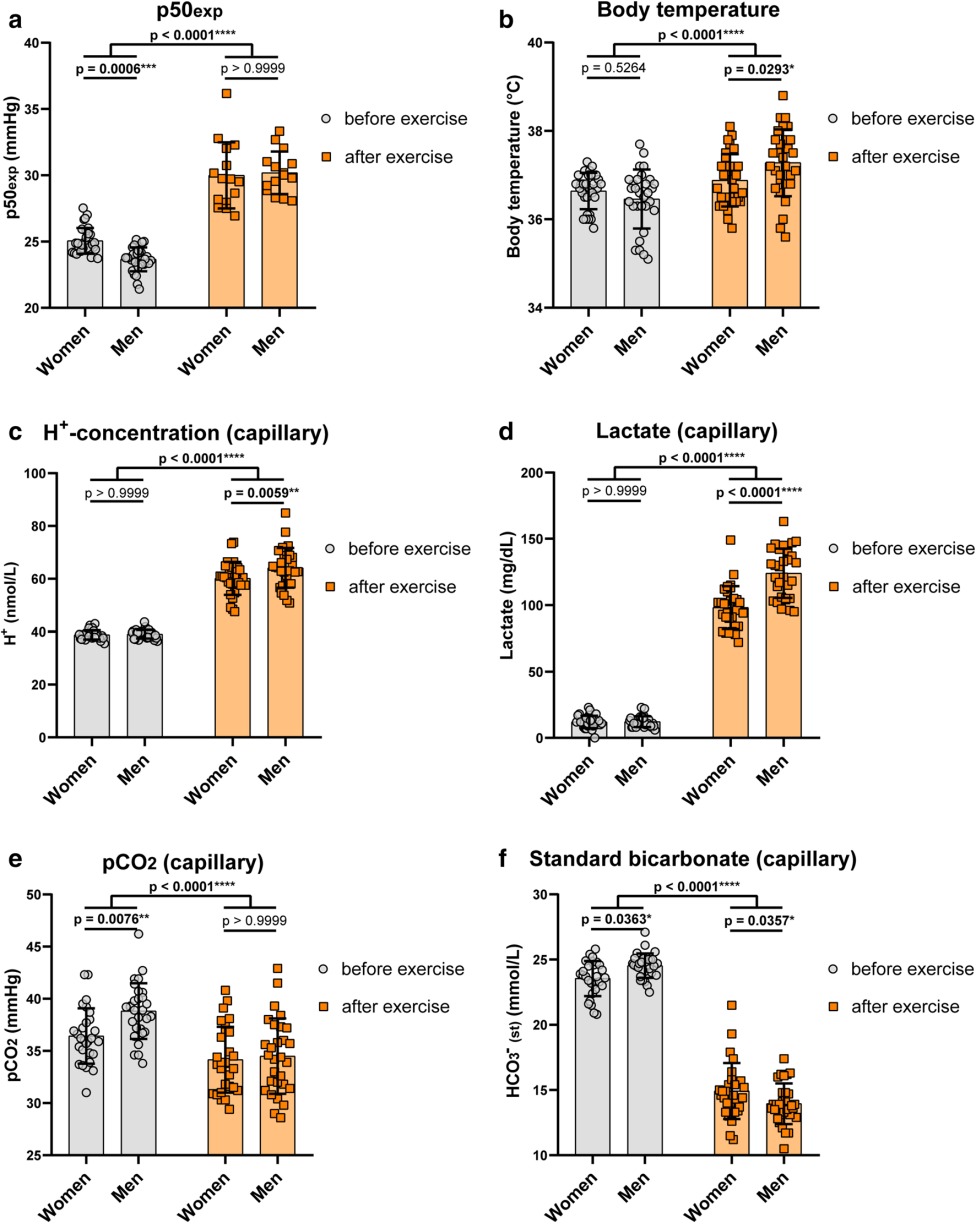
Experimental p50 and effectors of Hb-O_2_ affinity before and after exercise in women and men. (**a**) Oxygen affinity to haemoglobin is lowered during exercise to ensure that oxygen demand corresponds to cellular availability. Hb-O_2_ affinity is higher in men versus women at rest; however, after exercising this difference is nullified. Note: ODCs were recorded at isocapnic conditions and 37 °C, thus, p50 values are only attributed to pH in this setting. (**b**) Body temperature elevates during exercise, while men reached higher levels. (**c**, **d**) In vivo, at rest, capillary blood pH (**c**, as indicated by H^+^-level) and lactate level (**d**) were similar in both sexes. After VO_2_max test, men showed higher proton level (lower pH) that is explained by a stronger increase in lactate. (**e**, **f**) Capillary pCO_2_ (**e**) and standard bicarbonate (**f**) before and after exercise in women and men. (**a**) N = 30 for women and men before exercise; N = 15 after exercise. (**b**–**f**) N = 30 per group. Two-way ANOVA with post hoc Bonferroni test served to test for significance. Adjusted *p* values are indicated.

Before exercise, women showed a mean p50 of 25.09 ± 0.96 mmHg and after VO_2_max test of 30 ± 2.54 mmHg, indicating an ODC right shift at p50 of 4.91 mmHg on average (Fig. 7a).

In men, we found a p50 of 23.7 ± 0.9 mmHg at rest and a mean p50 of 30.26 ± 1.58 mmHg following VO_2_max test, indicating a right shift of ODC at p50 of 6.56 mmHg on average that is greater compared to women. The sex dependent Δp50 on average was 1.39 mmHg. No difference has been observed between women and men after the VO_2_max test (Fig. 7a). Thus, the greater right shift observed in men fully compensates for the higher affinity of oxygen to haemoglobin at rest and results from lower pH.

While alterations of ODCs before and after exercise were attributed to the pH only, blood gas analysis indicated that in vivo the alteration of Hb-O_2_ affinity following VO_2_max test is influenced by more factors. Although both sexes showed increased body temperature after exercise, body temperature in men was much more elevated (Fig. 7b), which also contributes to lower Hb-O_2_ affinity. Further, H^+^-level in men increased to a significantly higher level (causing lower pH) compared to women (Fig. 7c) that is attributed to a more pronounced lactate production in men (Fig. 7d). The blood pCO_2_ after exercise showed no difference (Fig. 7e). Interestingly, bicarbonate level strongly decreased during exercise (Fig. 7f), indicating the importance of the metabolic bicarbonate pool to buffer protons. Moreover, while bicarbonate level in men was higher compared to women at rest, after VO_2_max test, metabolic bicarbonate pool was lower in men (Fig. 7f).

### Venous but not capillary p50 estimates provide a good prediction of Hb-O_2_ affinity.

**Figure 8:**
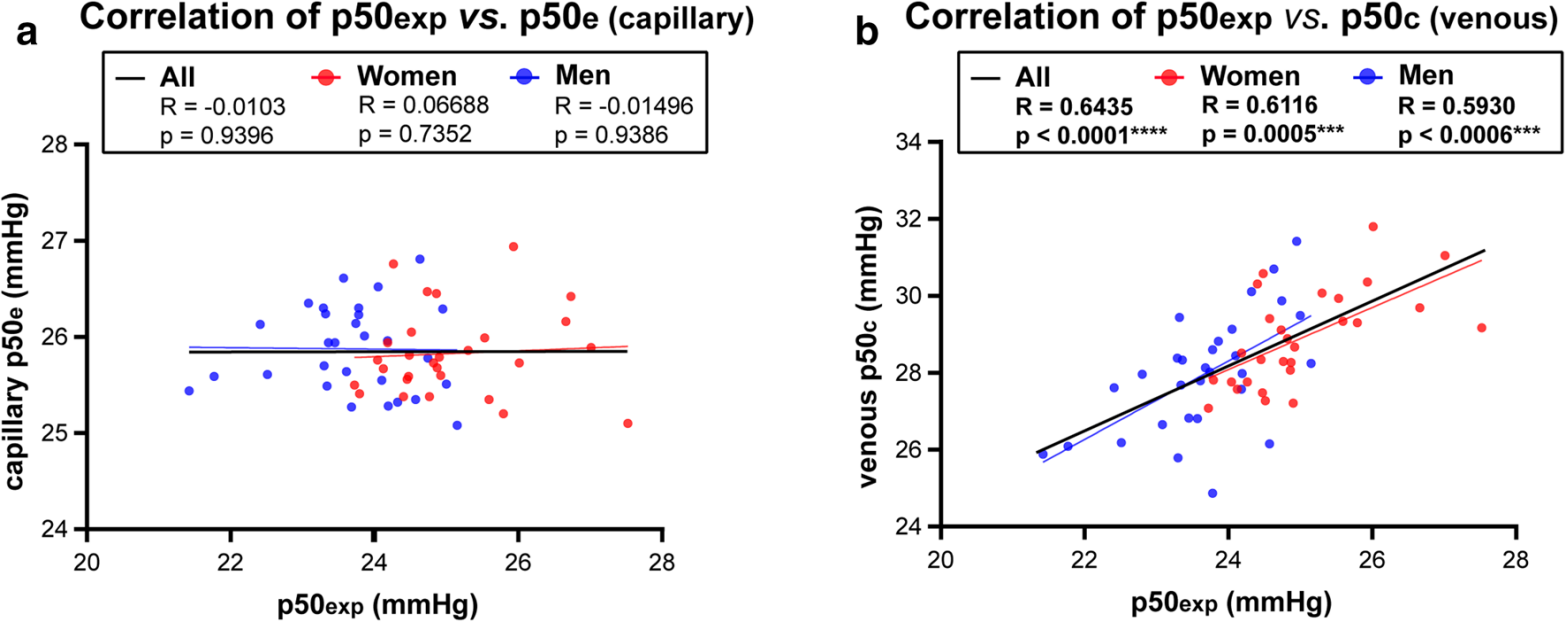
Correlation of experimental p50 (p50exp) values with predicted p50e/c values from blood gas analysis. (**a**, **b**) Correlation of p50 values as shown in Fig. 4 with estimated p50e values derived from capillary blood samples (**a**) and calculated p50c values from venous blood samples (**b**). The Pearson correlation coefficient R is indicated. Two tailed test with confidence interval 95% served to test for significance.

As p50 values derived from ODCs by tonometry need special equipment for measurements, it is crucial to know whether estimates from blood gas analysis provide sufficient information. We therefore correlated estimated (capillary) and calculated (venous) p50_e/c_ values with p50 values measured by tonometry (Fig. 8). We found no correlation, in neither women nor men, when correlating capillary blood samples, while venous probes indicated a good correlation.

## Discussion

Oxygen affinity to haemoglobin, as indicated by the p50 value, may be the most underestimated blood parameter in clinical practice. Moreover, potential influences of medication on cellular oxygen availability remains largely unknown. For instance, it has been shown that contrast media cause increased Hb-O_2_ affinity at normal pH and attenuated the Bohr effect in response to reduced pH^27,28^. This might be true for other compounds that interact with red blood cell membranes. Patients with high Hb-O_2_ affinity and/or reduced oxygen delivering capacity (anemia, hypoxemia) might therefore be at higher risk for tissue hypoxia, also during clinical treatments. By testing a group of 60 young, healthy volunteers, we show a marked diversity in baseline Hb-O_2_ affinity that is relevant for individual cellular oxygen availability.

One reason that Hb-O_2_ affinity is often neglected, might be that to accurately determine the p50, the ODC or its log-scale counterpart, a Hill plot, must be constructed^5^. P50 estimations from single blood samples have been shown to be misleading, especially at high oxygen saturation, which has been attributed to changes in shape of the ODC^29^. In our study, estimated p50_e_ values from capillary blood as well as calculated p50_c_ values from mixed venous blood were to be interpreted differently. By measuring ODCs and subsequent correlation analysis, it turned out that capillary p50_e_ values provide an insufficient prediction. We found a poor estimate for women and men as well. Of note, when correlating capillary p50_e_ values with capillary pCO_2_ values, a significant correlation was observed only for men but not women. In contrast, when correlating capillary p50_e_ values with capillary pH values, a very strong correlation was observed for both sexes. Thus, our data suggest that the estimation of p50_e_ is mainly based on blood pH, albeit the used Siggaard-Andersen Oxygen Status Algorithm^30,31^ includes the relevant parameters crucial for the estimation of ODC. We assume that if sO_2_ is above 97%, which corresponds to the upper flat part of ODC, the alteration of factors influencing the relationship between pO_2_ and sO_2_ will have only modest impact on the corresponding alteration of sO_2_. Thus, calculation of p50 from blood samples having an oxygen saturation of > 97% are likely to represent inaccurate estimates and, according to our data, cannot be recommended. In line with earlier assumptions^29^, we suppose that individual differences in the ODC shape are responsible for poor predictions of capillary probes.

In contrast, when correlating p50 values from ODCs with calculated p50_c_ values of venous blood samples, we found a significant correlation. Although individual values may differ, venous blood samples, thus, allow a much better prediction. These findings are in line with data using a Hemox-Analyzer, showing that estimates of p50 values based on venous blood gas analysis have a low bias compared to tonometry^32^. It has to be noted that these authors stated that despite a negligible bias, the precision of calculated venous p50 values was not sufficiently high to allow their use as substitutes for p50 values measured by tonometry in the clinical evaluation of polycythemia in individual patients^32^.

To address the issue of physiological differences in Hb-O_2_ affinity per se, we tested 60 volunteers that were either endurance exercise trained or not and of both sexes. Venous blood samples of all participants indicated p50_c_ values of 28.4 ± 1.47 mmHg (at pH: 7.35 ± 0.03 and pCO_2_: 49.35 ± 5.37 mmHg). Values derived from individual ODC’s indicated a p50 of 24.4 ± 1.17 mmHg (at pH: 7.43 ± 0.02; pCO_2_: 35.25 ± 0.7 mmHg; 37 °C). Standard deviation of p50 in both cases was slightly above 1 mmHg. However, when comparing lowest and highest measurements, we found a difference of Δp50_c_: 6.93 mmHg (calculated values from blood gas analysis; lowest p50_c_: 24.87 mmHg, highest p50_c_: 31.8 mmHg), which is supported by ODC derived values of Δp50: 6.11 mmHg. Thus, even in a cohort of young, healthy volunteers we found a marked diversity in Hb-O_2_ affinity.

We expected diversity in baseline p50 mainly attributable to fitness levels. In earlier reports, the right shift of ODC in athletes at rest has been attributed to higher 2,3-BPG level^33–36^. However, in line with other studies^12,37^, we found no influence of endurance exercise on Hb-O_2_ affinity.

Instead, sex differences mainly accounted for p50 diversity. In male blood, higher Hb-O_2_ affinity correlated with lower 2,3-BPG level and BPGM expression. In our study, the Δp50 of sexes was 1.39 mmHg. Earlier studies reported similar results^38,39^. This sex difference has been shown to exist only during sexual maturity, but not during pre-puberty or post-maturity^40^. Consequently, sex hormones have been postulated to influence 2,3-BPG synthesis and p50. However, only a weak or even no correlation has been found between testosterone/ estrogen and p50^40–42^. Our data add to observations of sex dependent differences in muscle fiber-type composition^43,44^, muscle myoglobin^45,46^ and the microcirculation^47^, all of which participate in cellular oxygen availability and utilization.

Another significant sex difference has been observed in blood pCO_2_, a further modulator of Hb-O_2_ affinity. Men showed higher capillary as well as venous pCO_2_ level than women. However, due to adequate higher bicarbonate levels, the pH was similar in all groups tested. Similar results have been reported for arterial pCO_2_, were a significant effect of sex was seen, with lower values for women (36.3 ± 2.9 mmHg) than men (41 0.2 ± 2.9 mmHg)^48^.

High bicarbonate level in men provide a better buffer capacity of the blood during exercise, allowing to compensate the greater lactate production. In addition, a higher haemoglobin concentration in men can bind more hydrogen ions. Moreover, higher haemoglobin level in men might be crucial to compensate the high Hb-O_2_ affinity, thus, low oxygen release to the tissue. We assume that cellular oxygen availability is similar in women and men at rest, due to a final balance in oxygen carrying capacity of the blood (Hb level) and the ability to release oxygen to the tissue (p50). Our data, thus, contribute to recent reports showing a correlation of Hb level and Hb-O_2_ affinity^49,50^.

Individual properties of Hb-O_2_ affinity are associated with an alteration of steepness of ODC. The slope of ODC represents one crucial feature of oxygen uptake (lung) and release (tissue). Our data show that a right shift of ODC is associated with lower slope of ODC. This has been observed following exercise mediated acidosis (increased hydrogen ions, Bohr-effect), as well as elevated 2,3-BPG levels (women vs. men at rest). When comparing p75, p50 and p25 values, the difference is always most pronounced in p75 and more modest in p25. This suggests that the steepness may have a stronger effect on oxygen uptake, while oxygen delivery is, relatively, less affected. It would be of interest to find p50 modifiers that cause a steeply sloping of ODC, as it would cause both, good O_2_-uptake as well as delivery.

When comparing the alteration in p50 due to maximum physiological exercise (VO_2_max test), we found a mean ODC right shift at p50 of 4.91 mmHg in women and of 6.56 mmHg in men. The strongest increase in p50 of all women was 10.37 mmHg (trained woman). The maximum p50 increase in men was 8.6 mmHg (untrained man). The alteration of p50 at maximum activity level may be seen as physiological tolerance, which is preserved only for minutes, when ATP demand exceeds its production by oxidative phosphorylation. Notably, the physiological difference in p50 (Δp50 at rest: approx. 6–7 mmHg), thus, lies only slightly below the range of maximum physiological tolerance (Δp50 between rest and VO_2_max: 8–10 mmHg). Individual baseline Hb-O_2_ affinity, therefore, is of huge importance. At rest, 2,3-BPG determinates the baseline p50 in healthy subjects, because of its strong correlation and the finding that pCO_2_ levels were in contrast to the observed Hb-O_2_ affinity. Thus, it is crucial and of clinical importance to understand the regulation of BPGM activity and expression.

Finally, it has to be noted that although oxygen release is crucial for cellular oxygen availability, elevated Hb-O_2_ affinity was shown to protect from hypoxia too. Some examples are 5-hydroxymethyl-2-furfural (5-HMF) and GBT1118 (a structural analog of GBT440) that represent allosteric effectors increasing Hb-O_2_ affinity and being beneficial in hypoxia^51–53^. This obvious paradox might be explained by the alteration of ODC slope in response to increased or decreased Hb-O_2_ affinity. As we have shown, the slope of ODC differs at rest, due to sex dependent differences in 2,3-BPG levels and during maximum exercise, due to changes in the blood pH. A left or right shift of ODC is most pronounced in the p75 and less in the p25 values. Therefore, with increasing Hb-O_2_ affinity, oxygen uptake is enhanced much stronger than oxygen release is impaired and, thus, elevated oxygen carrying capacity of the blood may fully compensate the high Hb-O_2_ affinity. Supporting, the beneficial effect of modifiers enhancing Hb-O_2_ affinity, were described in models of hypoxemia resulting from impaired oxygen uptake. We propose that oxygen transport capacity (Hb) and release (p50) are tightly balanced to ensure sufficient cellular oxygen availability. This is supported by findings that haemoglobin variants with high O_2_ affinity or mutations in the BPGM gene are clinically asymptomatic if the influence is mild, however, are associated with erythrocytosis at severe level^54–57^.

High Hb level causes increased blood viscosity and represents a risk factor for stroke and cardiovascular diseases. High Hb-O_2_ affinity is associated with elevated Hb level, as observed in women and men. Future studies are needed to better understand critical aspects of alterations of p50 at rest with the potential of more personalized medicine.

## Methods

### Recruitment of volunteers

Sixty healthy volunteers were recruited through online and in-print advertisements briefly outlining the study. Inclusion criteria were age (18 to 40 years), body mass index (18.5 to 25 kg/m^2^) and either being endurance trained (3 times per week for a minimum of 1 h per session since for at least one year) or untrained (no or only occasional sports activity). Upon volunteering for inclusion, potential participants received further detailed information with the option to ask questions, and reasonable time to decide whether to participate in the study. All volunteers gave their informed written consent. With the exception of 8 women using oral contraceptives and two women using iron preparation, the participants consumed no further drugs or nutritional supplements. The study was approved by the Charité Ethics Committee (EA1/154/18). All procedures and measurements complied with the Declaration of Helsinki (54th Revision 2008, Korea) regarding human subjects. Finally, four groups were selected by sex and exercise level (women trained, women untrained, men trained, men untrained), with N = 15 per group. Tests were performed in mixed order.

### Body composition

Body muscle- and fat mass were measured through multi-frequency bioimpedance analysis (Inbody 770, InBody Deutschland, Eschborn, Germany)^58^.

### VO_2_max test

Maximal oxygen consumption (VO_2_max) was determined on a calibrated motorized treadmill (H/P/Cosmos, Pulsar, Nussdorf-Traunstein, Germany) using an enhanced version of the Bruce protocol graded exercise test until each subject’s volitional fatigue^59^. The protocol was chosen as a performance test to be tolerated by the untrained participants of the study^60^. However, in order to further quantify the higher performance from the well-trained subjects, two more stages were added (Supplementary Table S1).

Oxygen consumption and carbon dioxide production were continuously measured from expired air using a breath-by-breath gas analysis system (Metalyzer 3B, Cortex Biophysik GmbH, Leipzig, Germany) connected to a computer for data-collection (Metasoft 3, version 3.9.9 SR1, Cortex Biophysik GmbH, Leipzig, Germany). The gas analysis system was calibrated using ambient air and verified gas concentrations of oxygen and carbon dioxide. The volume measurement system was calibrated using a 3-L Hans Rudolph calibration syringe (Cranlea Medical, Birmingham, UK). Heart rate was continuously recorded using a heart rate transmitter (Polar Electro Oy, Kempele, Finland) connected to the system. Subjects rated their perceived exertion (RPE) after 1 min into each stage according to the RPE-scale by Borg^61^. Upon reaching their individual maximum effort, subjects were verbally encouraged to continue the test for as long as possible^62^. All tests took place in the same air-conditioned laboratory under standardized conditions (19–22 °C ambient temperature, 99–102 kPa air pressure, 40–50% relative humidity) and were performed between 09 a.m. to 12 p.m. to avoid possible circadian influence on the test^63^. Subjects were advised to abstain from sleep deprivation the previous night as well as alcohol and drug consumption (except for oral contraceptives for the female subjects) and from caffeine consumption the morning of the test. During the test, all subjects wore light sportswear (T-shirt, sweatpants), the measurement equipment (mask, HR-transmitter), as well as a harness-system to prevent injury from falling. Immediately after cessation of the test, subjects walked to an adjacent room (distance 10 m) for blood-collection.

### Blood parameters

Upon arrival at the lab, subjects voided their bladder, had capsaicin-containing salve (Finalgon, Sanofi-Aventis, Frankfurt / Main, Germany) applied to their earlobe to increase local blood-flow for capillary blood collection^64^ and rested for 15 to 30 min. Capillary blood from the earlobe (approx. 100 µl) was then taken in a sitting position using a contact-activated lancet (BD Microtainer Blue, Becton and Dickinson, Franklin Lakes, NJ, USA), and venous blood (approx. 7 ml) was taken from the cubital vein using a 21-gauge Sarstedt-Multifly canula (Sarstedt AG, Nümbrecht, Germany). These blood collections were repeated immediately after the performance test. Capillary and venous blood samples were tested immediately using a Blood gas analyser (Radiometer ABL800 flex).

### Tonometry

Blood samples were arterialised via tonometry using humidified carbogen gas (95% O_2_, 5% CO_2_) at 37 °C, gently shaking. Deoxygenation was performed under identical conditions using a gas mixture of 95% N_2_, 5% CO_2_. 5% carbon dioxide corresponds to 38 mmHg pCO_2_ at sea level (760 mmHg), thus, represents the arterial pCO_2_ at 37 °C. Oxygen pressure (pO_2_) and blood oxygen saturation (sO_2_) were measured over time during de-oxygenation at isocapnic conditions via Blood gas analyser (Radiometer ABL800 flex) until blood oxygen saturation fell below 20%.

### Parameter calculation of oxyhaemoglobin dissociation curves (ODC)

The slope was determined for all oxyhaemoglobin dissociation curves (ODC) using the linear range of the Hill equation (20–60% sO_2_). Linear regression was performed according to the method of least squares and slope of the regression line was calculated. To determine p25, p50 and p75 values, polynomial regression for each ODC (20–85% sO_2_) was performed by the multiple least squares method and values were calculated using the regression function.

### 2,3-BPG

Measurement of 2,3-BPG in venous blood samples was performed using the 2,3-Diphosphoglycerate Kit (Roche Diagnostics, Mannheim, Germany, #10148334001) according to the manufacturer’s instructions and a 96-well Synergy HTX Plate Reader (BioTek Instruments, Winooski, USA) at 340 nm.

### Western blotting

To quantify BPGM protein level, full blood samples were lysed using a lysis buffer containing 50 mM Tris (pH 6, 8), 4 M urea, 1% SDS, and 12.5 mM DTT. Western blotting was performed as described previously^65^. Briefly, proteins of blood lysates were separated by SDS-PAGE and transferred to Hybond‐P membranes with BPGM protein being detected using a polyclonal rabbit anti-BPGM antibody (Novus Biologicals, Centennial, USA, #NBP1-86064). Following stripping with 0.2 mol/L NaOH, membranes were incubated with a polyclonal rabbit anti-GAPDH antibody (Acris Antibodies, Hiddenhausen, Germany, #BM439) to detect GAPDH protein as a loading control. Bound primary antibodies were visualized with horseradish peroxidase coupled secondary antibody (Santa Cruz Biotechnology, Dallas, USA, #sc-2030 and #sc-2031, respectively) and Western Bright Sirius substrate (Biozym Scientific, Hessisch Oldendorf, Germany) in an Intas ECL Chemostar Imager (Intas Science Imaging Instruments, Göttingen, Germany).

### Statistics

Statistical analysis was performed with the GraphPad Prism 8 software (GraphPad Software, San Diego, USA). To determine statistical differences between groups, two-way analysis of variance (ANOVA) coupled with a Bonferroni multiple comparison post hoc analysis was used. For correlation analysis, the Pearson's correlation coefficient (R) was calculated and a two-tailed Student's t-tests was performed to test for statistical significance. *P* values below 0.05 were considered significant.

## Supplementary information

Supplementary Figures.

## Data Availability

For original data (including de-identified individual participant data), please contact michael.faehling@charite.de.
