# Supplementary material for: A broad diversity in oxygen affinity to haemoglobin
Source: Sci Rep. 2020 Oct 9;10:16920. doi: 10.1038/s41598-020-73560-9 (PMC7547706; doi:10.1038/s41598-020-73560-9)
Supplement: Supplementary file 1 — Supplementary Figures. [file 41598_2020_73560_MOESM1_ESM.pdf]

A broad diversity in oxygen affinity to haemoglobin

Björn Balcerek[1#], Mathias Steinach[2#], Julia Lichti[1,2], Martina A. Maggioni[2,3], Philipp N. Becker[1], Robert Labes[1], Hanns-Christian Gunga[2], Pontus B. Persson[1], Michael Fähling[1*]

[1]Institut für Vegetative Physiologie, Charité – Universitätsmedizin Berlin, corporate member of Freie Universität Berlin, Humboldt-Universität zu Berlin, and Berlin Institute of Health, Berlin, Germany;

[2]Institut für Physiologie, Zentrum für Weltraummedizin Berlin (ZWMB), Charité – Universitätsmedizin Berlin, corporate member of Freie Universität Berlin, Humboldt-Universität zu Berlin, and Berlin Institute of Health, Berlin, Germany;

[3]Department of Biomedical Sciences for Health, Università degli Studi di Milano, Milan, Italy

[#] B.B. and M.S. contributed equally to this study.

* Correspondence:

Michael Fähling, Charité – Universitätsmedizin Berlin, Institut für Vegetative Physiologie,

Charitéplatz 1, D-10117 Berlin, Germany

Email: michael.faehling@charite.de; Phone: 0049 30 450 528263

SUPPLEMENT

Supplementary Table S1: Applied Test Protocol according to the Bruce protocol.[1] 1 MET (Metabolic Equivalent) equals the basal metabolic rate and an oxygen consumption of 3.5 ml/ kg body weight/ min. Thus, increases in oxygen consumption and energy expenditure can be expressed as manifolds of “MET”.[2]

* Note: 24 % was the maximum grade of the treadmill.

Stage	Duration (min)	Speed (km/h)	Grade (%)	≈ MET
Standing	3	0	0	2
1	6	2.7	10	5
2	9	4	12	7
3	12	5.4	14	10
4	15	6.7	16	13
5	18	8	18	16
6	21	8.8	20	18
7	24	9.6	22	20
8	27	10.4	24	22
9	30	11.2	24*	24

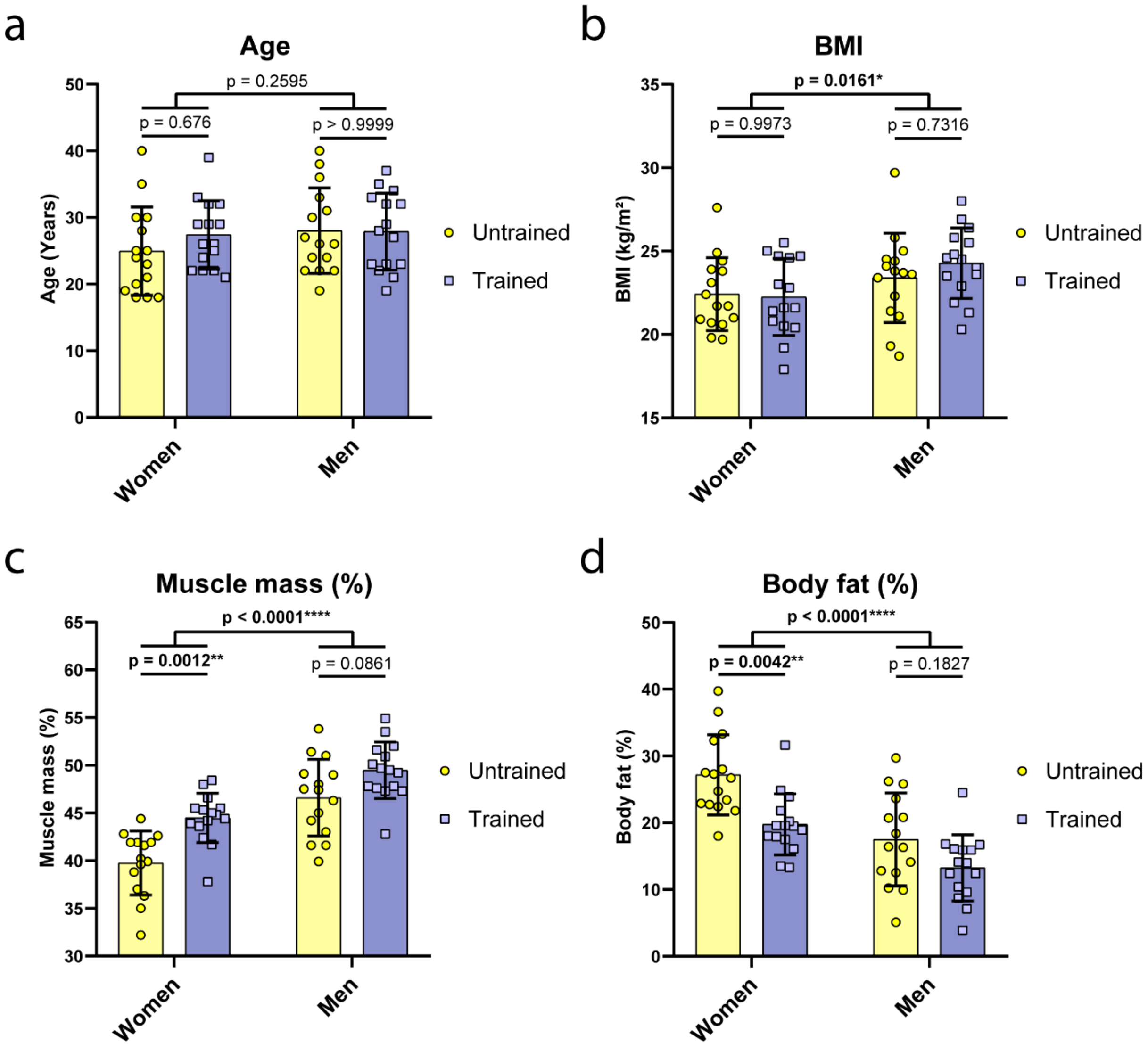

Supplementary Figure S1: Basic parameters of groups tested (complement to Figure 1). We tested 60 volunteers that were of both sexes and either trained or untrained. **a:** Participants of the groups were of similar age (average 27 years) and **b:** showed a relatively comparable body mass index (BMI), albeit men were slightly higher than women. **c, d:** Muscle mass percentage was higher in trained groups and in men *vs*. woman (C) that corresponds to body fat (D). N = 15 per group. 2way ANOVA with post hoc Bonferroni test served to test for significance. Adjusted p-values are indicated.

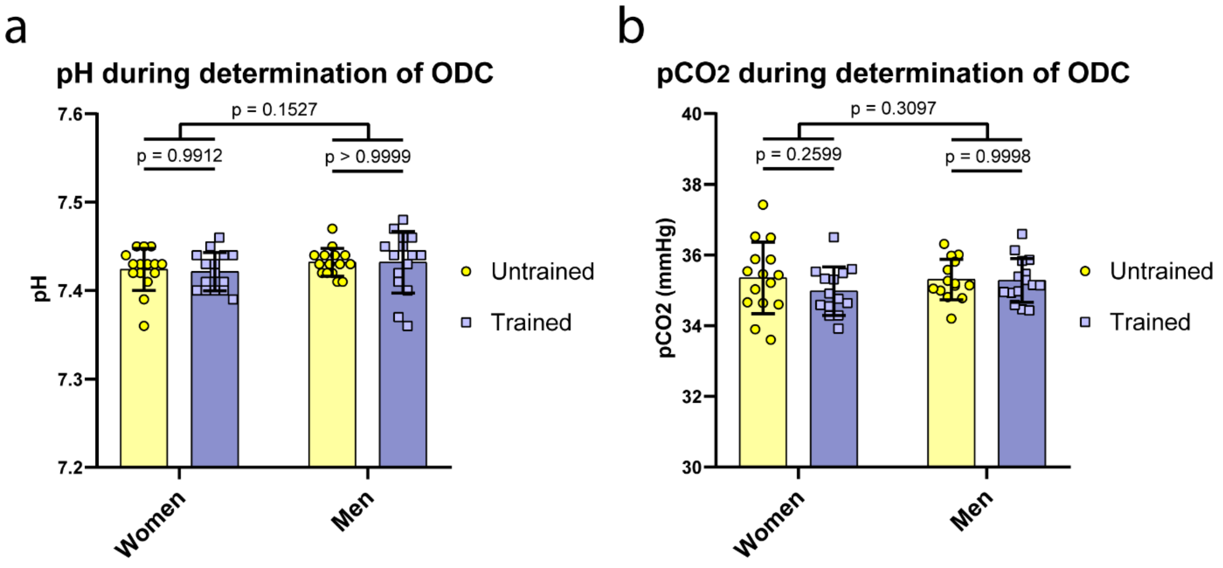

Supplementary Figure S2: Standardised experimental conditions for the determination of ODCs. pH (**a**) and pCO_2 (**b**) of blood samples were kept stable during measurements by fixed pCO_2 (5 % in overflowing gas) and temperature (37°C) to ensure comparability of all samples. N = 15 per group. 2way ANOVA with post hoc Bonferroni test served to test for significance. Adjusted p-values are indicated.

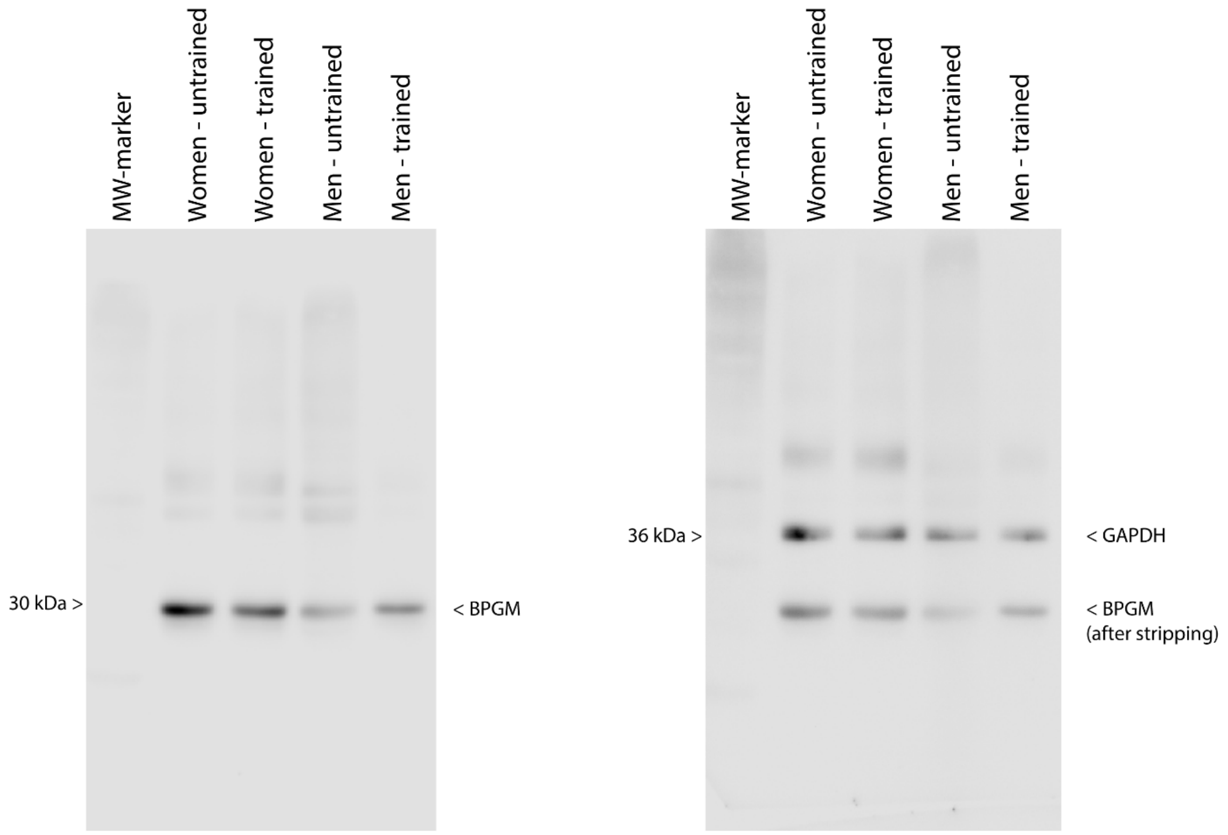

Supplementary Figure S3: Original Western blots detecting BPGM and GAPDH from a pool of human blood samples as shown in Figure 5.

SUPPLEMENTARY REFERENCES

1 Bruce, R. A., Blackmon, J. R., Jones, J. W. & Strait, G. Exercising Testing in Adult Normal Subjects and Cardiac Patients. *Pediatrics* **32**, SUPPL 742-756 (1963).

2 Ainsworth, B. E. *et al.* Compendium of physical activities: classification of energy costs of human physical activities. *Med Sci Sports Exerc* **25**, 71-80, doi:10.1249/00005768-199301000-00011 (1993).
